# Efficacy and safety profiles of manidipine compared with amlodipine: A meta-analysis of head-to-head trials

**DOI:** 10.3109/08037051.2010.518670

**Published:** 2010-09-29

**Authors:** FLORENT F RICHY, STEPHANE LAURENT

**Affiliations:** 1 Department of Public Health, Epidemiology and Health Economics, University of Liege Faculty of Medicine, Belgium; 2 Department of Pharmacology, Pompidou European Hospital, INSERM U 970, and Paris Descartes University, Paris, France

**Keywords:** Ankle oedema, calcium antagonists, hypertension, meta-analysis

## Abstract

The aim of this meta-analysis was to compare the efficacy and safety profile of manidipine 20 mg with that of amlodipine 10 mg. A systematic research of quantitative data produced or published between 1995 and 2009 was performed. Head-to-head randomized controlled trials (RCTs) of 12 months minimum duration reporting comparative efficacy (changes in systolic and diastolic blood pressure) and safety (total adverse events and ankle oedema), were included. Four high-quality RCTs, accounting for 838 patients (436 received manidipine and 402 received amlodipine) were included. The effi cacy of manidipine and amlodipine was statistically equivalent: effect size for DBP =−0.08 (*p* = 0.22) and SBP =−0.01 (*p* =0.83).The global safety of manidipine was signifi cantly better than amlodipine: the relative risk (RR) for adverse event was 0.69 (0.56 – 0.85), and particularly for ankle oedema RR was 0.35 (0.22 – 0.54). Publication bias was not signifi cant and the robustness of the analyses was good. These data suggest a better efficacy/safety ratio of manidipine over amlodipine.

## Introduction

Hypertension is a major risk factor for myocardial infarction and the most important modifiable risk factor for stroke (1,2). Hypertension is also the most important risk factor for disability-adjusted life-years and mortality in developed countries and lower mortality in developing countries (3). In addition, hypertension severely impacts the quality of life among patients (2,4). Despite all benefits demonstrated in response to blood pressure lowering, hypertension management remains suboptimal among western populations (1,5). The reasons have been repeatedly analysed (2), among which the side-effects of antihy-pertensive drugs emerge as an important issue in clinical practice.

Dihydropyridine calcium-channel blockers (CCBs) are potent antihypertensive agents. Their vasodilatory effects are associated with adverse effects (AEs) such as peripheral oedema, headache and flushing (6). Ankle oedema is a common adverse event observed during treatment with CCBs, and mainly dihydropyridines, and is dose related (6,7). The three mechanisms put forward to explain the formation of ankle oedema after CCBs are the arte-riolar vasodilation, the impairment of the local vascular autoregulation of blood flow and the impaired protection against hydrostatic load (7). This adverse event has an early onset, since it is observed during the first 2 weeks of treatment, is dose dependent and is more frequent in elderly patients (7).

In several cases, ankle oedema is responsible for treatment discontinuation or limited patient's compliance to anti-hypertensive treatment and has a deleterious impact on health-related quality of life. Manidipine, a third-generation CCB characterized by high lipophilicity and vasoselective action, has demonstrated a better tolerability profile than short-acting calcium antagonists requiring multiple daily doses (e.g. nifedipine, felodipine), delayed or modified-released formulations (e.g. nifedipine) and agents with longer half-life (e.g. amlodipine). Indeed, in clinical pharmacological studies using the pretibial subcutaneous tissue pressure technique (7) and foot-ankle volume measurement (5), ankle oedema was less frequent with manidipine than with amlodipine, felodipine and nifedipine.

Ankle oedema is related to the baroreflex-induced activation of the sympathetic system, constricting the post-capillary venules, thus reinforcing the pressure gradient at the capillary level, which originates from pre-capillary arteriolodilatation and post-capillary venoconstriction (7). Hydrostatic pressure aggravates the phenomenon of capillary transudation (7). Manidipine proved to induce less ankle oedema than other dihydropyridines, probably because of a lower degree of sympathetic activation (8-11).

The objective of the present study was to compare the efficacy and safety profile of manidipine with that of amlodipine, currently the most prescribed dihydropyridine. Several randomized parallel groups clinical studies concluded to a similar or even better antihypertensive efficacy of manidipine, compared with amlodipine, with less frequent side-effects (8-11). We used a meta-analysis of randomized controlled trials (RCTs), since the statistical power of each trial is rather limited and cannot by itself provide a clear-cut estimate of the relative efficacy and safety of manidipine compared with amlodipine.

## Methods

A systematic research of any controlled trial containing relevant data was performed using validated methods (Cochrane), followed by peer review, data extraction and quality scoring blinded for authors and data sources. An exhaustive systematic search has been performed, using a maximum of sources (Medline, Premedline, Embase, Cochrane controlled trials register, manual review of the literature and congresses abstracts). Inclusion criteria were: RCT, duration of 1 month at least, amlodipine as comparator, assessment of efficacy of systolic and diastolic blood pressure reduction, global side-effects and ankle oedema using validated techniques, publication range January 1995 to July 2009. Leading authors were contacted for unpublished data. The publications retrieved were discussed for methodological standards and inclusion compatibility by two separate authors. We did our best to provide as robust assumptions as possible. To this end, analyses were performed using professional dedicated software in a conservative fashion. The number of patients presenting with the researched outcomes were preferentially used against the number of events (12).

Dichotomical outcomes were expressed as relative risk (*RR = Risk_exposed_/Risk_non-exposed_*) or risk differences (*RD = Risk_exposed_–Risk_non-exposed_*). Continuous outcomes were expressed as standardized mean differences (i.e. effect sizes): (*ES = Mean_exposed_ – Mean_non-exposed_*/[(*Std*(*Mean*)]_exposed, non-exposed_).

The level of statistical significance was set at 5% for association and 10% for heterogeneity. The intergroup difference was assessed on the basis of heterogeneity over 10%. The global and individual estimators were surrounded by their 95% confidence intervals. Publication bias (the bias related to the preferential publication of trials reporting significant efficacy) was exhaustively investigated using funnel plot representation and formal assessments involving Fail Safe N, regression intercept method and rank correlation approach (13). All operations were performed using a registered copy of Comprehensive Meta-Analysis 2.2.040 (Biostat Inc, USA) (14).

## Results

Four studies, accounting for 838 patients exposed to either to manidipine 10–20 mg or amlodipine for 12–48 weeks were ultimately included in the meta-analytic process (8–11) (Table I).

**Table I:** Characteristics of the retrieve comparative trials on manidipine vs amlodipine.

Author (ref.)	Design	Population, *n* inclusion	Intervention (dose)	Duration (weeks)	Losses to follow-up	Outcomes
Zanchetti et al. 2001 (11)	DB, MC, R	35–70 years; manidipine = 245; amlodipine = 244; 95 < DBP < 115; SBP < 200 after w/o; M = 183; A = 178	Manidipine (10–20 mg); amlodipine (5–10 mg)	w/o 3–48	26%	SBP, M = − 17.5; A = − 19.6; DBP, M = − 13.7; A = − 14.1; ankle oedema, M: 20/245; A = 52/244; total AE, M = 75/245; A = 99/244
Coca et al. 2006; MAISH (8)	DB, MC, R	Manidipine = 99; amlodipine = 96; Over 60 years; DBP < 90; SBP > 145	Manipidine (10–20 mg); amlodipine (5–10 mg)	w/o 2–12	9.2%	SBP, M = − 19.5; A = − 18.4; DBP, M = − 5.2; A = −4.9; ankle oedema, M: 4/99; A = 4/96; total AE, M = 23/99; A = 27/96
Martinez-Martin et al. 2005; AMANDHA (9)	R	Manidipine = 61; amlodipine = 30; diabetes II; HT; nephropathy; reninangiotension blockers > 6 mo	Manipidine (20 mg); amlodipine (10 mg)	24	n/a	SBP, M = − 19.1; A = − 12.7; DBP, M = − 7.7; A = − 10.9; ankle oedema, M: 2/61; A = 8/30; total AE, M = 5/61; A = 8/30
Martinez-Martin et al. 2009; MARIMBA (10)	DB, R	Manidipine = 32; amlodipine = 32; metabolic syndrome	Manipidine (20 mg); amlodipine (10 mg)	12	1.56%	SBP, M = − 15.9; A = − 16.2; DBP, M = − 5.2; A = − 4.9; ankle oedema, M: 1/32; A = 3/32; total AE, M = 1/32; A = 6/32

DB, double-blinded; R, randomized; MC, multicentric; w/o, wash-out period; DBP, diastolic blood pressure; SBP, systolic blood pressure; AE, adverse events.

### Efficacy: office diastolic and systolic blood pressures

Amlodipine and manidipine reported a statistically similar efficacy in the reduction of diastolic and systolic blood pressure. Effect sizes were −0.085 (−0.22 to 0.092) and −0.015 (−0.15 to 0.12), respectively (Table II). On average, systolic blood pressure was reduced by 18.3 and 17.3 mmHg, and diastolic blood pressure was reduced by 8.5 and 10.5 mmHg after manidipine and amlodipine, respectively.

**Table II:** Meta-analysis of the blood pressure lowering effect in response to either manidipine or amlodipine during head-to-head studies.

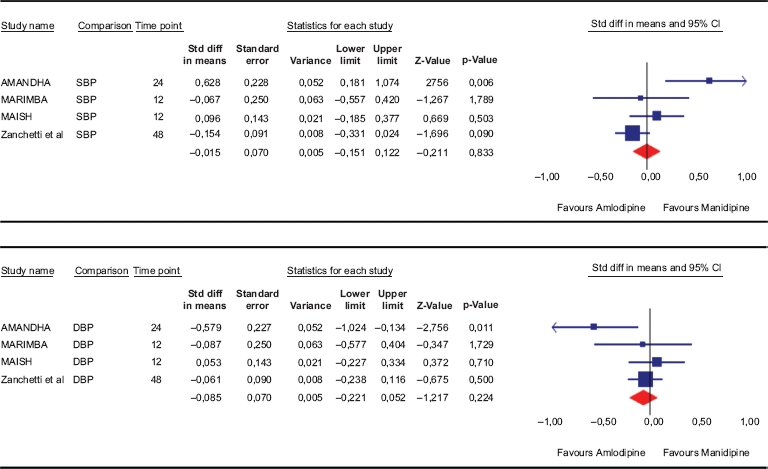

SBP, office systolic blood pressure; DBP, office diastolic blood pressure. Mean differences (and standard errors) are displayed.

### Safety: total AEs

The relative risk for developing any AEs when allocated to manidipine was significantly lower [RR = 0.69 (0.56–0.85);*p* = 0.001] than after amlodipine. In terms of risk difference, a significant 11% (5–17%) reduction of the risk for adverse event was computed in favour of manidipine (Table III).

**Table III:** Meta-analysis of the adverse effects (AEs) in response to either manidipine or amlodipine during head-to-head studies, expressed either as risk ratio (RR, upper part) or risk difference (RD, lower part).

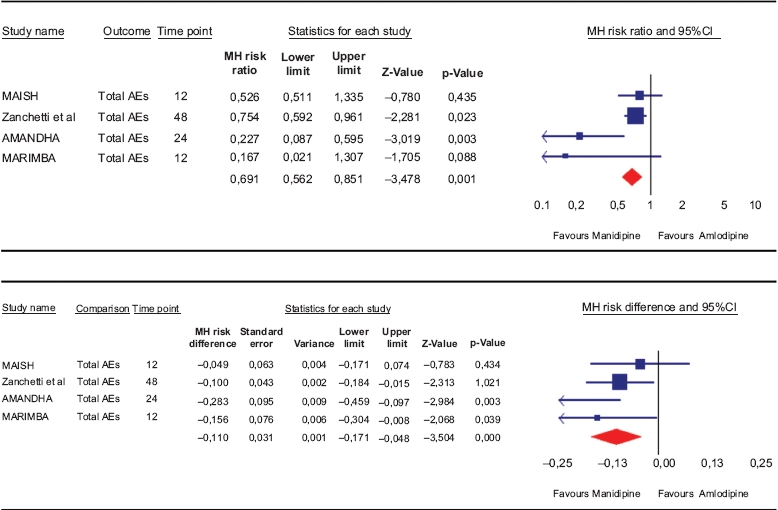

MH: Mentel-Haenszel method.

### Safety: ankle oedema

The pooled relative risk for developing ankle oedema was 0.35 (0.23–0.54) for manidipine against amlodipine. In terms of risk difference, a significant 11.3% (7–16%) reduction of the risk for ankle oedema was computed in favour of manidipine (Table IV).

**Table IV:** Meta-analysis of ankle oedema in response to either manidipine or amlodipine during head-to-head studies, expressed either as risk ratio (RR, upper part) or risk difference (RD, lower part).

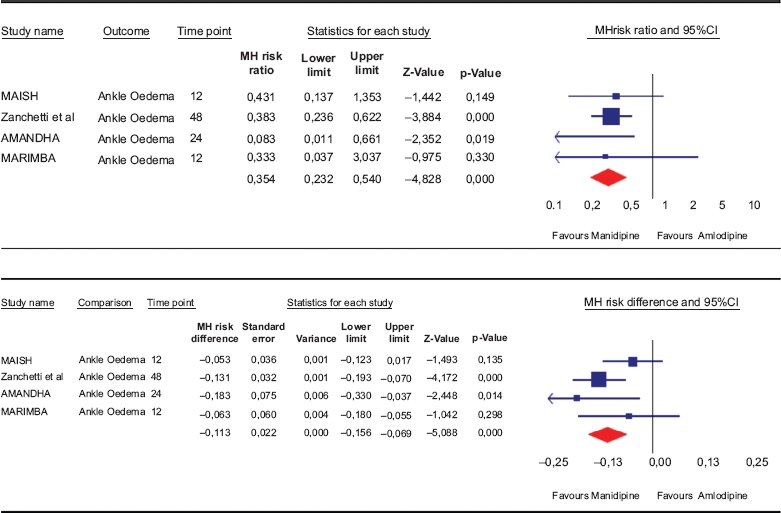

MH: Mentel-Haenszel method.

### Publication bias

**Figure 1:**
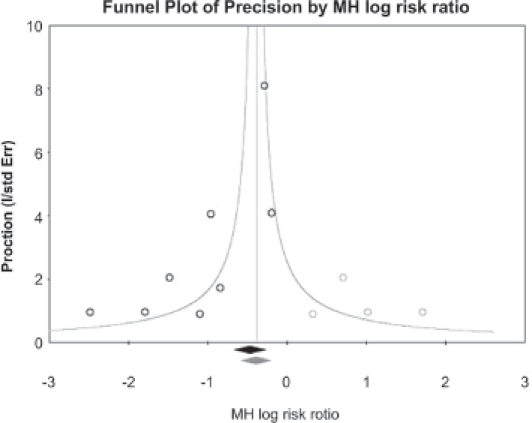
Publication bias assessment and robustness testing: funnel plot with a Duval and Tweedie Trim and Fill sensitivity analysis. This graph plots the log RR against the studies precision (open circles), as well as the log global estimate (black lozenge). The assumption of no publication bias is linked to a symmetrical distribution of the studies on the left and right sides of the global estimate (which shows an inverted funnel). In case of asymmetry, Duval and Tweedie set up a method that allows for simulating the “missing” studies (open grey circles) that make the graph symmetrical. When including these dummy studies, a new global estimate can be recomputed (grey lozenge) for comparison with the actual global estimate. Table V provides the global estimates with and without correction. What can be seen is that whatever the correction applied (as a sensitivity analysis), the estimates remain significant, showing that no significant publication bias affects the dataset.

No evidence of significant publication bias was found in the dataset. When adjusting the safety analysis of total AEs and ankle oedema for potential publication bias, the point estimate remained signifi cantly in favour of manidipine, suggesting a good robustness of the analyses (Table V and Figure 1).

**Table V:** Duval and Tweedie Trim and Fill sensitivity analysis.

		Fixed effects			Random effects			
	Studies trimmed	Point estimate	Lower limit	Upper limit	Point estimate	Lower limit	Upper limit	Q value
Observed values		−0.46829	−0.65665	−0.27993	−0.76498	−1.19516	−0.33480	17.72861
Adjusted values	4	−0.39147	−0.57423	−0.20872	−0.46422	−0.90358	−0.02485	29.62359

Explanations are given in the fi gure legend.

## Discussion

The present study demonstrated that, despite a similar antihypertensive efficacy, manidipine 10 – 20 mg was associated with significantly less total AEs in general, and ankle oedema in particular, compared with amlodipine 5 – 10 mg in the long-term management of mild to moderate hypertension in patients aged over 35 years.

The high rate of uncontrolled blood pressure remains a major issue in most countries (2). The causes have been analysed in several reviews (15), among them poor persistence with therapy (16,17), most often related to patient intolerance for AEs. Although dihydropyridine CCBs are powerful anti-hypertensive agents, their vasodilatory effects are associated with AEs such as peripheral oedema, headache and flushing (6). Pharmacokinetic and pharmacodynamic difference between dihydropyri-dines could lead to a lower incidence of vasodilation related AEs with the third-generation molecules, like manidipine, compared with second-generation molecules like amlodipine. Amlodipine is particularly interesting to compare, since this is the most pre-scribed dihydropyridine in most countries.

Our working hypothesis was that ankle oedema would occur to a lesser extent after manidipine than after amlodipine, despite a similar antihypertensive efficacy. Indeed, although all dihydropyridines CCBs have been developed to reach similar BP lowering efficacy despite different potency, they may differ in sympathetic activation. The high lipophilicity and vasoselective action of manidipine could represent an advantage regarding baroreflex activation, know as a key element in the pathophysiology of ankle oedema. It is generally accepted that in response to blood pressure lowering after dihydropyridines, the baroreflex-induced activation of the sympathetic system leads to contraction of post-capillary venules, which in turn increases the pressure gradient at the capillary level. Hydrostatic pressure aggravates the phenomenon of capillary transudation (7). In the meantime, pre-capillary arteriolodilatation, a direct effect of dihydropyridines, protects against the vasoconstrictive effects of sympathetic activation (7,18,19).

Recent pharmacodynamic studies have shown that manidipine induced a lower degree of sympathetic activation than amlodipine, which in turn could explain the lower incidence of ankle oedema. In a randomized crossover trial, with a 4-week placebo run-in period at baseline and a 4-week placebo wash-out period between the 16-week treatment periods, Fogari et al. (19) demonstrated that, compared with amlodipine, manidipine was associated with signifi cantly smaller increases in morning and evening ankle volume. Plasma norepinephrine levels were not significantly altered during the 16-week manidipine treatment period, but significantly increased during the corresponding amlodipine treatment period.There was a significant positive correlation between changes in norepinephrine levels and ankle volume during amlodipine therapy, particularly in the morning, but this was not the case during manidipine therapy.

In the present study, we demonstrated that, despite a similar antihypertensive efficacy, manidipine 10–20 mg was associated with significantly less ankle oedema, compared with amlodipine 5–10 mg, in the long-term management of mild to moderate hypertension.

Several limitations should be discussed, as with all meta-analyses. Although carrying a high level of evidence, modern meta-analyses may be biased by selective publication of positive trials. The present data-set was tested for robustness against this assumption and no significant difference in the overall estimates could be found while adjusting for potential publication bias. RCTs can be limited in their ability to identify rare adverse events, as they are not sufficiently powered. Post-marketing authorization studies can allow regulatory bodies and pharmaceutical industries to detect safety signals; however, their ability to draw inferences against comparators is very limited because of the heterogeneity of patients included. In this perspective, meta-analysis allows for more precise comparison of the relative risks of adverse events, as they keep the randomization scheme of the original studies. Heterogeneity was present in efficacy outcomes, although it was nonsignificant in safety outcomes. This can be attributed to the differences in inclusion criteria, leading to variability in response, although the relative safety of manidipine against amlodipine appeared to be stable. A random effect combination model was used to account for this, according to current guidelines.

In conclusion, these data suggest a better efficacy/safety ratio of manidipine over amlodipine. These results may have an important impact, since patients who persists with treatment have better chance of normalizing their blood pressure, thus reducing their risk of cardiovascular and renal events.

## Notice of correction

[ePub ahead of print] 14 October 2010, DOI: 10.3109/08037051.2010.518670. The Early Online version of this article published ahead of print on 14 October 2010 contained an error in the abstract where SBP was shown as −0.08 instead of as −0.01. The corrected sentence is shown in this issue. Some errors were also shown in the labeling of the tables. The corrected versions are shown in this issue.

## References

[1] Lloyd-Jones D, Adams R, Carnethon M, De Simone G, Ferguson TB, Flegal K (2008). American Heart Association Statistics Committee and Stroke Statistics Subcommittee. Heart Disease and Stroke Statistics – 2009 Update. A Report from the American Heart Association Statistics Committee and Stroke Statistics Subcommittee. Circulation.

[2] Mancia G, de Backer G, Cifkova R, Dominiczak A, Fagard R, Germano G (2007). Guidelines for the Management of Arterial Hypertension. The Task Force for the Management of Arterial Hypertension of the European Society of Cardiology (ESC) and of the European Society of Hypertension (ESH). J Hypertens.

[3] Ezzati M, Lopez AD, Rodgers A, Vandeer Horn S, Murray CJL, the Comparative Risk Assessment Collaborative Group (2002). Lancet.

[4] Coyne KS, Davis D, Frech F, Hill MN (2002). Health-related quality of life in patients treated for hypertension: A review of the literature from 1990 to 2000. Clin Ther.

[5] Bramlage P, Thoenes M, Kirch W, Lenfant C (2007). Clinical prac-tice and recent recommendations in hypertension management-reporting a gap in a global survey of 1259 primary care physicians in 17 countries. Curr Med Res Opin.

[6] Opie LH (1988). Calcium channel antagonists. Part IV: Side effects and contraindications drug interactions and combinations. Cardiovasc Drugs Ther.

[7] Fogari R (2005). Ankle oedema and sympathetic activation. Drugs.

[8] Coca Payeras A, Sladek K, Lembo G, Alberici M (2007). Antihyper-tensive efficacy and safety of manidipine versus amlodipine in elderly subjects with isolated systolic hypertension: MAISH study. Clin Drug Investig.

[9] Martinez-Martin F, Rodriguez-Rosas H (2005). Relationship between sympathetic activation, pulse pressure and heart rate in hypertensive type 2 diabetic patients with microalbuminuria, treated with manidipine vs. amlodipine. J Hypertens.

[10] Martinez-Martin F (2009). Manidipine in hypertensive patients with metabolic syndrome: The MARIMBA Study. Expert Rev Cardiovasc Ther.

[11] Zanchetti A, Omboni S, La Commare P, De Cesaris R, Palatini P (2001). Efficacy, tolerability, and impact on quality of life of long-term treatment with manidipine or amlodipine in patients with essential hypertension. J Cardiovasc Pharmacol.

[12] Cohen J (1988). Statistical power analysis for the behavioral sciences.

[13] Sterne JA, Gavaghan D, Egger M (2000). Publication and related bias in metaanalysis: Power of statistical tests and prevalence in the literature. J Clin Epidemiol.

[14] Borenstein M, Hedges L, Higgins J, Rothstein H (2005). Compre-hensive meta-analysis, Version 2. Englewood Cliffs.

[15] Simons LA, ortiz M, Calcino G (2008). Persistence with antihypertensive medication: Australia-wide experience. Med J Aust.

[16] Jones JK, Gorkin L, Lian JF, Staffa JA, Fletcher AP (1995). Discontinuation of and changes in treatment after start of new courses of antihypertensive drugs: A study of a United Kingdom population. BMJ.

[17] Van Wijk BL, Klungel OH, Heerdink ER, de Boer A (2005). Rate and determinants of 10-year persistence with antihypertensive drugs. J Hypertens.

[18] Fogari R, Zoppi A, Corradi L, Preti P, Malalamani GD, Mugellini A (2000). Effects of different dihydropyridine calcium antagonists on plasma norepinephrine in essential hypertension. J Hypertens.

[19] Fogari R, Malamani GD, Zoppi A, Mugellini A, Viscardi A, Lastoria C (2000). Manidipine has less oedematigeneous potential than amlodipine [abstract]. J Hypertens.

